# Population attributable fractions continue to unmask the power of prevention

**DOI:** 10.1038/s41416-018-0062-5

**Published:** 2018-03-23

**Authors:** Freddie Bray, Isabelle Soerjomataram

**Affiliations:** Cancer Surveillance Section, International Agency for Research on Cancer, Lyon, France

## Abstract

The population attributable fraction is a critical driver of evidence-based cancer prevention. With an increasing recognition of the need for high-level investment in cancer control, there is an overwhelming need for a new generation of descriptive studies that globally promote the long-term public health and economic benefits of cancer prevention.

In planning and prioritising cancer prevention strategies, the population attributable fraction (PAF) is established as an indispensable quantifier of the number of new cancer cases (or deaths) attributable to key modifiable risk factors. Brown and colleagues in this issue of the *British Journal of Cancer* provide estimates of the proportion of new cancers attributable to such risk factors in the UK in 2015.^1^ Nearly 4 in 10 cancers are attributed to the determinants included in the study, representing 135,000 new cancer cases. Tobacco smoking remains the most important cause of cancer, followed by being overweight or obese, which is consistent with previous estimates from 2010.^2^ The authors indicate that many of the most commonly diagnosed cancers, including lung and melanoma, can be directly attributed to the majority (>70%) of risk factors included in this updated study, highlighting the vast potential to reduce the cancer burden through directed primary prevention actions.^1^

The overall PAF estimate for the UK of 38% is in line with the recently reported 2014 estimate of 42% for the US,^3^ but it is higher than the figure of 33% published in Australia for 2013.^4^ These discrepancies may be partially explained by differentials in the subset of risk factors selected for inclusion in the respective studies, and in the underlying prevalence of exposure between nations. For example, a few environmental factors (air pollution, ionising radiation, and occupational exposures to carcinogens) were included in the most recent UK exercise, but were not included in the Australian equivalent. Despite these methodological differences, there is consistency across the studies in the array of major contributors to cancer incidence (differences between studies in parentheses), which include: tobacco smoke (13–18%), dietary factors (5–7%), overweight (4–8%), infections (3%), alcohol (3–6%), and UV radiation (4–6%). The absolute proportions and the relative ordering of the factors have minor variations, for example, solar UV radiation is the second leading cause of cancer incidence in Australia,^4^ whereas overweight/obesity was ranked second in both the US^3^ and the UK.^1^

Taken together, the results underscore the extent to which tobacco remains the leading cause of cancer incidence in high-income settings.^1,3,4^ The UK study, which also provides PAF estimates for the constituent countries of the UK, reports a somewhat larger overall PAF in Scotland (41%), compared with England (37%); differences in smoking prevalence between the two nations likely contribute to this finding, with 18% of cancers ascribed to smoking in Scotland, relative to 15% in England. Given the well-documented social and economic inequalities linked to tobacco use, as noted by Brown et al.,^1^ the estimation of PAF in different societal groups can yield evidence to support tobacco control interventions targeted at vulnerable populations. With further regional variations seen in overweight/obesity, dietary factors, infections, and occupational carcinogens, a comprehensive package based on local evidence is needed. One that targets emerging risk factors and guides the implementation of proven population-based interventions is likely to pay the greatest dividends in reducing the future cancer burden.^5^

In addition, if 40% of cancers diagnosed today in high-income populations could potentially be avoided by eliminating exposure to known lifestyle and environmental risk factors, what might we say for low-income and middle-income countries, which are facing an ever-greater annual cancer burden?^6^ Recent global PAF estimates for infection,^7^ obesity,^8^ and UV radiation^9^ emphasise the need to tailor cancer control actions in accordance with localised patterns of risk factors and cancer incidence.^10^ It is also worth highlighting that 1 in 30 cancers are attributed to infectious agents in the aforementioned high-income countries; however, this is likely to be closer to 1 in 3, or even 1 in 2 cancers diagnosed in parts of Sub-Saharan Africa, where infectious agents remain a central cause of cancer.^7^

Cancer now ranks as the first or second cause of premature death in almost 100 countries, according to WHO estimates for 2015.^11^ Thankfully, there is an increasing global recognition of the need for high-level investment in cancer control, alongside other major non-communicable diseases (NCDs). Building on the UN Sustainable Development Goals and the specific target of a one-third reduction in premature mortality from NCDs by 2030, a new cancer resolution was unanimously adopted by 192 governments at the World Health Assembly in May 2017.^12^ The resolution notes the potential for different cancer control actions to prevent around one-half of all cancers, through the promotion of highly effective strategies for cancer prevention that are locally relevant to communities worldwide. In addition, the resolution links with the updated Appendix 3 of the *WHO’s Global Action Plan for the Prevention and Control of NCDs 2013–2020*,^13^ which provides policymakers with a list of cost-effective and affordable interventions to address the cancer burden. The key primary prevention interventions include implementing the tobacco control policies outlined within the WHO *Framework Convention on Tobacco Control* (notably increasing excise taxes and prices on tobacco products), and vaccination against human papilloma viruses and hepatitis B virus.

The inherent diversity in the major risk factors for cancer in different world regions, coupled with constant changes in their prevalence and distribution, points to the need for regular and systematic quantifications of PAF as critical drivers of evidence-based cancer prevention. The lack of availability of such robust data is at present, however, a major challenge. The need for governments to build population-based systems of data collection to inform cancer control, particularly in low-income and middle-income countries, is unambiguously stated in the World Health Assembly’s cancer resolution. Though our current knowledge indicates that a substantial proportion of cancers are preventable and prevention is cost-effective, actions must be promoted and implemented. The extended latency of cancer means impact from prevention measures tends to be seen decades rather than years after the intervention, requiring politicians and planners to take a longer-term view of the clear public health and economic benefits.^14,15^ From this standpoint, there is an overwhelming need for a new generation of descriptive studies that promote the enduring impact of prevention. A great start would be to demonstrate through scenario-building prediction models, such as the recent Nordic example showing the impact on cancer from reductions in overweight/obesity levels,^16^ how effective, in terms of impact and cost, interventions can be in reducing the burden and suffering from cancer in the near-term and longer-term future, nationally and worldwide.
